# HSN—helping in mental distress: a new emotion focused psychological first aid program

**DOI:** 10.3389/fpubh.2025.1589608

**Published:** 2025-06-25

**Authors:** Marina Scheele, Sandra Appel, Isabelle Rausch, Martin Schecklmann, Jakob Klein, Susanne Staudinger, Roger Schmidt, Peter M. Kreuzer, Berthold Langguth

**Affiliations:** ^1^Department of Psychiatry and Psychotherapy, University Regensburg, Regensburg, Germany; ^2^Study Center for Evangelical Youth Work Josefstal, Josefstal, Germany

**Keywords:** psychological first aid, mental health, HSN, interpersonal emotion regulation, emotion-focused, helping in mental distress, mental health education, “Helfen in Seelischer Not”

## Abstract

Psychological first aid programs aim to train lay people to help others who are experiencing a mental health crisis or developing mental health problems. The current article introduces the new German psychological first aid training program HSN. The acronym HSN stands for “Helfen in Seelischer Not” (helping in mental distress) but also for the action chain “hear, speak, network.” The HSN program differs from other well-examined mental health first aid programs by (1) focusing primarily on emotions instead of disorders, (2) using a blended approach, combining face-to-face and online elements right from the start of the project and (3) a low-threshold approach with a course duration of two hours. Here we specify the HSN concept and report first results from a sample of 150 students. The participating students completed a self-report questionnaire before and after the course regarding their “confidence to help.” The program demonstrated good feasibility without any relevant side effects. On a descriptive level we observed an improvement of the averaged values of all questionnaire items after the course. These positive initial results merit the further development of HSN as a low-threshold psychological first aid program.

## 1 Introduction

The prevalence of mental disorders is high and further increasing, while access to professional help services is often difficult. Several factors may play a role, among them the stigma of mental disorders, which is associated with reduced help-seeking (1, 2), a general lack of knowledge and understanding of mental health problems or insufficient knowledge about access routes to help services (3). To fill these gaps, psychological first aid programs have been developed with the goal to teach the general population about mental health literacy and improve the competence of lay persons to deal with people who experience mental health problems. Substantial evidence suggests that such programs like Mental Health First Aid (MHFA) are associated with positive changes in knowledge, attitudes, behavioral intent and reductions in stigma (4, 5). Besides MHFA several other programs such as Psychological First Aid (PFA) (6) or YAM (Youth Aware of Mental Health) have been developed and scientifically evaluated (7). However, recent systematic reviews conclude, that the evidence for the efficacy of these programs remains limited due to a lack of studies with good quality and highlight the need for further research in this field (8, 9).

These mentioned first aid trainings typically involve information transfer about the symptoms of various mental disorders or syndromes, with a specific emphasis on the recognition of crisis situations. This requires a considerable amount of time as there exist many relevant mental diseases (e.g., depression, bipolar disorder, anxiety disorders, obsessive compulsive disorder, posttraumatic stress disorder, personality disorders, schizophrenia, alcohol and drug dependency, anorexia, dementia, just to name the most common ones). The duration of psychological first aid courses in turn represents a major access barrier. This means that those who do not have the time or capacity to attend a long-lasting course, may not participate in such programs. Therefore, we developed a new psychological first aid program with a shorter timeframe with the name “HSN – Helfen in Seelischer Not (Helping in mental distress).” In the past, there have already been pilot trials to design shorter psychological first aid trainings of common programs with auspicious outcomes related to attitudes, confidence and practical skills in early intervention of depression and suicide-prevention (10–12). The HSN concept differs from other programs by its emotion-focused approach providing specific action strategies for different emotional states of the concerned person irrespective of a potential diagnostic framework. Consequently focusing on the leading emotion of the concerned person should give the first responder the advantage of being able to apply the acquired knowledge more universally to different situations independently from specific psychiatric disorders.

In the last years numerous studies have shown that emotions and emotion regulation abilities are closely linked to psychopathological symptoms (13). Difficulties with emotion regulation are associated with various mental disorders (14), whilst successful emotion regulation leads to good health outcomes, improved relationships, good work performance and general well-being (15–19). Compared to *intrapersonal* emotion regulation, *interpersonal* emotion regulation strategies have received less attention in clinical-psychological research so far. However, interpersonal emotion regulation has shown beneficial effects on stress reduction, affect and general well-being (20–23). Based on these findings the HSN concept focuses on interpersonal emotion regulation of the three basic emotions fear, anger and grief/sadness—three emotions that are consistently identified as core basic emotions in major theoretical models (24). They serve essential adaptive functions—such as threat detection (fear), boundary setting (anger), and signaling loss or social disconnection (sadness). But these affective states are not only evolutionarily adaptive but also highly relevant in the onset and progression of psychological crises and mental disorders (25–31). That’s why these three emotions are repeatedly used in the analysis of emotion regulation processes, for example in the FEEL-E questionnaire (32) or the FEEL-KJ questionnaire (33, 34). The “triage assessment system for crisis intervention” (TAS) also focuses on the affective domains anger/hostility, anxiety/fear and sadness/melancholy (35).

Besides this emotion-focused approach, another characteristic of the HSN concept is the blended-learning format: other first aid programs are originally designed as face-to-face courses and have been updated by e-learning and blended learning versions with good effects in recent years (36–38). The HSN program uses a blended approach consisting of a face-to-face course complemented by online content right from the start. Both components will be explained below.

## 2 Pedagogical framework(s)

**Figure 1:**
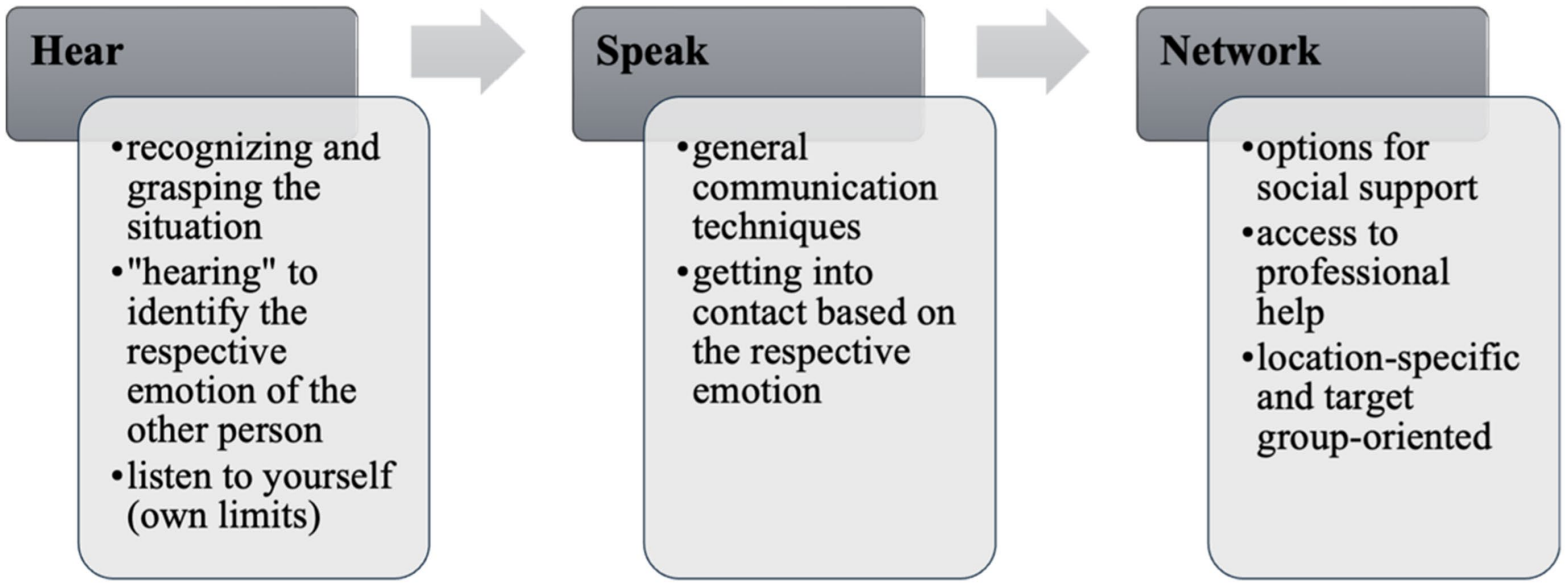
The HSN step sequence.

The letters HSN do not only stand for “Helfen in Seelischer Not” (Helping in mental distress) but are also a memory aid for the three steps of the program which are “Hinschauen” (hear), “Sprechen” (speak) and “Netzwerken” (network) (see Figure 1).

Other programs also use such abbreviations as a reminder, for example PFA with the step sequence LLL (Look-Listen-Link) (6). While these concepts are often based on knowledge of psychological disorders, the HSN concept is based on the three basic emotions that often occur in crises: fear, anger and grief/sadness. In the “hear” module (Hinschauen), participants are first taught how to identify the respective emotions (e.g., facial expressions, physical reactions). The next step is to work out what function the respective emotion has and what underlying need it points to (motive of the emotion). Strictly speaking, this is fundamental psychoeducation on emotions. The emotion-focused psychoeducation in HSN aims to improve recognition and differentiation of emotions (own and those of others), which in turn provides orientation how to help. Another aspect of the module “hear” is learning to look on yourself as a first responder, also from an emotion-focused perspective. In a study about gender differences in empathy during adolescence and the role of emotional self-awareness it was found that in girls, difficulties identifying their own feelings can negatively influence the ability to differentiate between ones’ and others’ emotions, which leads to more self-focused and aversive reactions when confronted with others’ suffering (39). On the one hand, we are using this part to increase self-awareness about factors that prevent people from helping. On the other hand, we want to convey, that it is important to respect the own well-being and the personal limits when helping others. Therefore, reflecting the own emotional state is equally important as detecting the leading emotions of the person in distress. The leading emotion of the person in distress provides guidance for the second step, the “speak” part (Sprechen).

**Figure 2:**
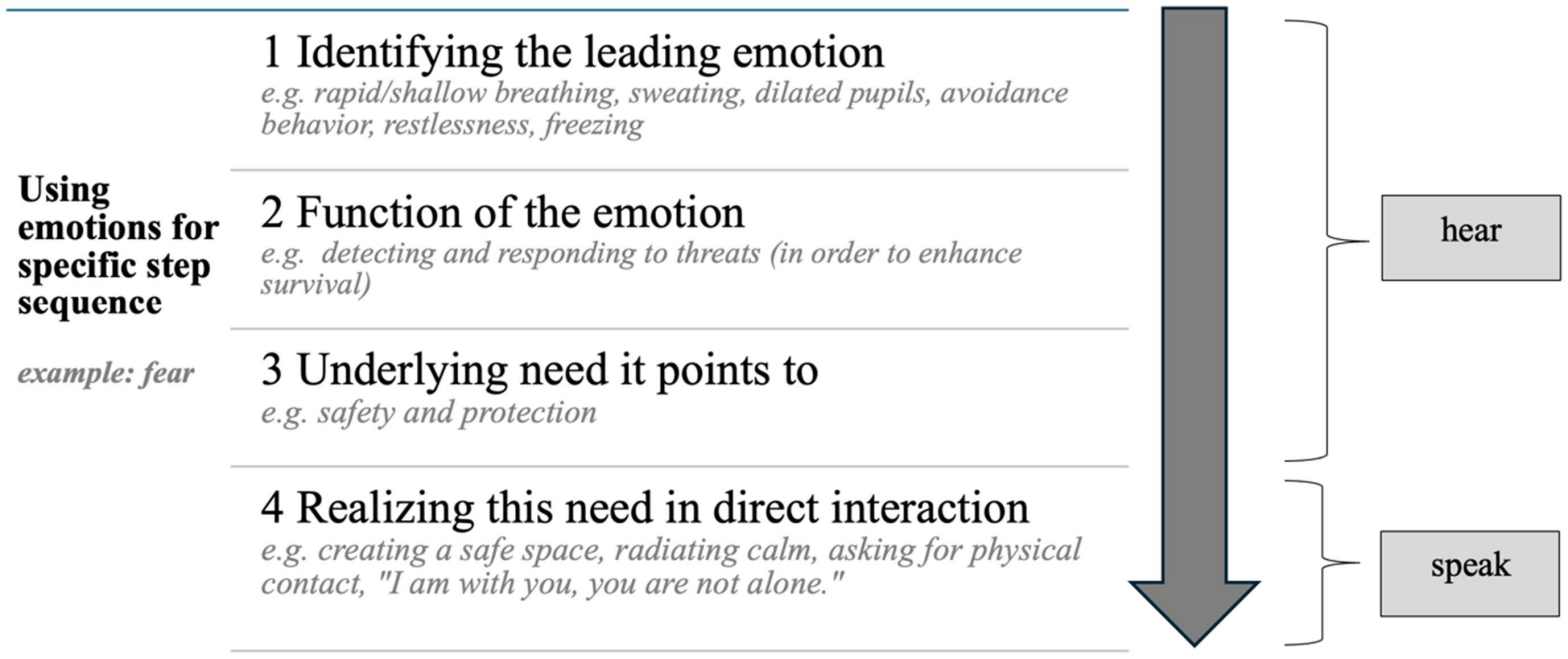
Specifying the action chain with the emotion-focused approach.

In this “speak” part, general communication techniques (e.g., “taking the concerned person and their problems seriously”) are combined with recommendations for specific contact strategies which are based on the leading emotions. Participants learn to interpret the emotion as the expression of an individuals’ need (e.g., fear signals the presence of danger), and how they can respond to this need in the direct interaction. This includes the setting (e.g., creating a safe place), non-verbal communication (e.g., going to eye level) as well as exemplary sentences (see Figure 2). During the entire training, exercise sequences and interactive elements are repeated to practice this process of identifying the leading emotion together with the underlying need and then to address this need in direct contact with individual situations of the participants. Participants are encouraged to suggest example situations for this interactive training part. In addition, the facilitator provides specific recommendations for detecting and dealing with suicidal tendencies, as all participants should acquire competencies in the application of the three steps hear – speak – network in the context of suicidal behavior.

The last module “network” focuses on the fact that the first aider is not solely responsible for the entire process—equivalent to classical first aid courses where the first responders are taught seeking assistance from bystanders and calling the ambulance. Together with the participants, options for social support (e.g., family, friends, teachers) as well as access to professional help (e.g., psychotherapists, crisis hotlines or suicide prevention lifeline) are explored. It is a special characteristic of the HSN concept that context-and local-specific contact points are provided during the training. It was noticed, that although many people are generally aware of the help services (e.g., “We can contact the school psychologist”), they do not know specific people and addresses and how to get in contact with them (“Who exactly is this person at your school?”). This gap should be bridged by presenting local offers and explaining how to get in contact with them.

## 3 Learning environment

The learning goals of the HSN program are to strengthen skills in dealing with people in mental crisis and to train specific courses of action as a first aider. For this purpose, very basic knowledge of psychological mechanisms in crisis situations, competencies how to deal with them and knowledge of help services should be expanded. The overall aim is to strengthen self-confidence and self-efficacy as a helping person.

The face-to-face courses are delivered by a qualified HSN-facilitator. They do not need to have any previous professional experience in the psychological or medical field. Instead, the project aims for facilitators with various professional and social background in a peer-related context to conduct the courses in their individual social spheres of activity, e.g., an employee who leads the courses with his/her colleagues. This ensures that the facilitators have a good knowledge of the individual topics of their target groups. The facilitator training program is designed to ensure a high standard of course delivery through a structured, multi-phase approach. It consists three key components: First, prospective facilitators participate in a three-day, in-person training seminar, during which they are introduced to the course concept, its pedagogical foundation, and relevant methodological and didactic adaptations. A central element of this seminar is the opportunity for self-reflection and self-experience in the role of a facilitator. Participants actively explore group leadership by practicing facilitation and engaging with simulated challenging group situations, which are then discussed and reflected upon within the group setting. Following the seminar, facilitators-in-training enter an observation phase, during which they attend at least two real-life HSN courses conducted by experienced facilitators. This phase allows them to deepen their understanding of the course structure and group dynamics by linking observed experiences with the theoretical knowledge gained during training. After this, each trainee independently facilitates a full course and reflects on the experience in a written case report. This report serves as the basis for a final certification meeting, during which the trainee’s experiences, practical reflections, and theoretical understanding are reviewed and discussed. In addition, relevant knowledge is formally assessed. Upon successful completion of this process, the individual is certified as an HSN facilitator. To maintain certification and ensure ongoing quality assurance, facilitators are required to conduct at least four courses per year and participate in quarterly supervision sessions. These supervisions provide space for reflection, skill development, and the continued professional growth of facilitators within a structured peer setting.

The target group of the HSN course program includes the entire population starting with the age of 14. This means that the project runs courses in German schools from the eighth grade onwards. No psychological knowledge or prior experience of the participants is required, so it can be realized in almost all contexts like companies, sports clubs or religious institutions. In order to be particularly low-threshold, the courses last around two hours, which enables integrating them into everyday work or school life. In all groups (10 to 20 participants), the courses take place in a circle or semi-circle of chairs to foster interaction within the group. The facilitators use a standardized PowerPoint presentation to implement the basic structure of the HSN course program. Frontal teaching is minimized whereas active participation and interaction among the participants is encouraged. Similarly like in group therapy (40) participants are reminded about confidentiality at the beginning of the course. After a short introduction of the emotion-focused approach and the “hear – speak – network” action chain concept, the facilitators illustrate its application in concrete situations proposed by the participants. Initially, the plan was to develop group-specific training programs for different target groups. However, during the test phase it became clear that it is very important to discuss the topics and concerns raised by the course participants and that these topics were largely independent from group characteristics such as age or group context. We therefore expanded the group-specific focus to the extent that each course is individually adapted depending on the needs of the participants that arise during the course. That is why the HSN-facilitators are taught different pedagogical (interactive) methods as part of their own instructor training to work with the groups as targe-group-oriented as possible. Participants of the courses should be encouraged to share their own experiences. The action chain steps shown above (hear, speak, network) are supplemented by symbols as memory aids. These symbols were—metaphorically speaking—packed one after the other into a first aid kit to use them as important tools for psychological first aid.

In recent years, a number of studies have shown that blended learning (addition of digital learning content) is as effective as the traditional face-to-face method with some advantages like improvement in retaining knowledge, skills acquisition, patient outcomes, and cost saving (41). In a systematic review and meta-analyses, there are also indications in which blended learning has proven more effective on knowledge and skills compared with non-blended learning and have shown a positive impact on mental health (42). The HSN concept wants to adapt these findings into the psychological first aid training. So, after such a face-to-face course, participants are given access to the digital HSN learning portal. The platform offers a range of interactive, case-based learning materials that allow users to apply and deepen their understanding of the course content at their own pace. Central to the platform are several animated case vignettes that depict realistic psychosocial situations (e.g., a neighbor showing signs of suicidality). For each vignette, users can engage with a variety of interactive learning activities, such as true-or-false statements, multiple-choice questions, and scenario-based tasks (e.g., “What would be an appropriate way to respond in this situation?”). In addition, each vignette is accompanied by an in-depth expert video, in which experienced psychologists analyze the situation, apply the course’s core intervention model (HSN action sequence), and reflect on key challenges and nuances. The platform concludes with a comprehensive self-assessment quiz, enabling users to review and test their understanding of the key concepts covered. This blended learning approach is intended to promote knowledge retention, support transfer to everyday practice, and encourage active, self-directed learning beyond the classroom setting.

## 4 Assessment and results to date

### 4.1 Methods

The HSN project is continuously evaluated to provide sound evidence of its effectiveness, to ensure quality standards and to adapt the course program if necessary. For this reason, participants are asked to provide demographic data and to answer several course related questions at three time points: before the face-to-face course (pre), immediately after the course (post) and 6 months or 1 year after the course (follow-up). Here we present first preliminary results from a sample of social work students.

### 4.2 Participants

159 social work students participated in HSN courses, which were held at their university. Participation was voluntary. 9 participants did not complete the post questionnaire, resulting in data from 150 participants (mean age 25.23 ± 7.86 years, 120 female, 30 male).

### 4.3 Design

Due to its exploratory character the pilot study employed a single-group pre-post design. Data were collected at two times: before (pre) and right after the course (post). Thus, the evaluation only refers to the face-to-face course, but not to the digital component.

### 4.4 Instruments

Participants were asked to complete a self-report questionnaire. It included basic sociodemographic items (e.g., age, gender) and ten statements assessing confidence to help others in challenging or crisis situations.

The items were developed specifically for this study with the purpose of a first evaluation of the particpants’ experience newly developed program. Item content was drawn conceptually from common established instruments in the field (43) and items were reviewed by experts in psychological first aid to ensure content relevance. Each item was rated on a five-point Likert scale ranging from 1 = strongly disagree to 5 = strongly agree. The ten items are provided in Supplementary Table S1. According to the purpose of the study we opted for this form of descriptive course evaluation with specifically customized questions instead of using standardized psychometrically validated questionnaires.

### 4.5 Procedure

As part of the academic curriculum of the “Social Work” degree program, HSN courses were held by an certified HSN facilitator and as outlined in the previous chapter. A total of 10 courses were held, each lasting 120 min. Participants completed the questionnaires before and after the course.

### 4.6 Data analysis

The statistical program R Studio was used for the analysis. Due to the pilot character of the study and the relatively small sample size, we limited the analysis to descriptive statistics. All analyses were performed using R Studio. For each item on the confidence to help scale, the mean (M) and standard deviation (SD) were calculated both pre-and post-course. As part of the descriptive analysis, participants were categorized according to whether they had improved, worsened or remained unchanged on the given item, and the frequencies for each category were calculated. Thus, the focus was on providing a descriptive overview of the participants’ self-reported changes in confidence.

## 5 Results

Descriptive data demonstrate an increase in agreement for all questions. Comparing the pre-and post-course scores to describe participants’ overall confidence levels, in all items changes in confidence were found. For the questions about how to handle an emotional crisis situation (item 2), approaching the person and inviting them to a conversation (item 8), referring them to another help service (item 9) and what to do with expressions of suicide (item 10) we found the largest effects. It is notable that the greatest increase was observed in questions referring to the sections of “speak” and “network” (see Table 1).

**Table 1:** Descriptive data of the questions asked pre/post HSN training.

Items	*M* pre (SD)	*M* post (SD)	*N* improvement	*N* unchanged	*N* deteriorated	*N* total
1) I know what an “emotional crisis” is.	4.00 (0.742)	4.63 (0.550)	84	62	4	150
2) Meeting a person in mental distress, I know how to handle it.	3.11 (0.894)	4.20 (0.543)	109	38	3	150
3) Meeting a person in mental distress, I can put myself in their place.	3.97 (0.685)	4.21 (0.609)	41	102	7	150
4) Meeting a person in mental distress, I observe them more closely.	4.31 (0.657)	4.69 (0.517)	59	86	5	150
5) Meeting a person in mental distress, I can trust my gut feeling that something is off or unusual.	4.11 (0.640)	4.38 (0.631)	43	103	4	150
6) Meeting a person in mental distress, I can take good care of myself at the same time.	3.31 (1.003)	3.93 (0.791)	78	66	6	150
7) Meeting a person in mental distress, I can also be aware of my own feelings.	3.49 (0.903)	3.97 (0.737)	64	80	6	150
8) Meeting a person in mental distress, I know how to approach them and invite them to a conversation.	3.21 (0.980)	4.26 (0.660)	105	39	6	150
9) Meeting a person in mental distress, I know how to refer them to another helper or support service.	3.07 (1.206)	4.37 (0.661)	111	35	4	150
10) Meeting a person who expresses to me that they want to take their own life, I know what to do.	2.85 (1.228)	4.17 (0.660)	111	37	2	150

## 6 Discussion

### 6.1 Summary and interpretation

In the current article, the new German training program for psychological first aid (HSN) is introduced and initial results of its evaluation are presented. Relevant characteristics of the HSN concept are the low-threshold access and the use of an emotion-focused highly interactive approach. A meta-analysis showed that such programs with interactive elements, in particular role-playing and simulation-based scenarios, enable greater training effectiveness (44). In the field of first aid programs, HSN with its low-threshold approach fills the gap to complex courses lasting some hours to several days, such as “Mental Health First Aid” (42). The extent to which short courses can bring about change at all is repeatedly discussed critically in the literature (45). The descriptive results of our evaluation indicate that HSN program participants consider themselves as more confident to help others immediately after the course. This goes in line with the results of previous studies, which have found strong evidence of improvement in participants’ confidence in helping others with a mental health problem (43, 46–51). The results of the systematic evaluation correspond to the experience of the HSN-facilitators. Participants interacted actively during the courses and reported spontaneously that the course helped them to increase their confidences to act in emotional crisis situations. Feedback often emphasizes the emotion-based approach because it has a depathologizing and destigmatizing effect. Emotional needs and interpersonal interactions are treated as an aspect of everybody’s life and not considered as a symptom of a disorder. This enables participants to apply what they have learnt in a variety of contexts, both if an emotional crisis occurs in the context of a psychiatric disorder or as a (non-pathological) response to a major life event.

### 6.2 Limitations of the results

The presented results should be interpreted with caution as they derive from preliminary data collected from a non-representative sample. The study sample consisted of students of social work (predominantly women of a young age), who presumably have higher interest and more prior knowledge about psychological first aid than the average person. Additionally, the assessment of course effects was focusing on the subjective experience of participants. The follow-up survey for this sample is not available, so no conclusions can be drawn about the sustainability of the increase in confidence. The influence of the digital learning platform, which is part of the course concept and accessible only after course participation, can only be assessed with the follow-up survey. Future studies are necessary for a comprehensive evaluation, which then should include a control group, validated questionnaires and follow-up assessments, not only assessing the subjective “confidence to help” but also quality and quantity of help.

### 6.3 Implications and conclusion

All in all, the HSN concept represents a new psychological first aid program. With its short duration of 2 h and its blended learning approach, it aims at low-threshold access to fill the gap between purely online based offers and existing courses of longer duration. Further characteristics are the emotion-focused concept and the inclusion of interactive elements. The presented preliminary results suggest that this concept is feasible and can increase the “confidence to help” of participants. Next steps include the evaluation of the course in larger and more representative samples with more comprehensive and standardized assessment instruments, and in comparison to both waiting list control groups and other psychological first aid programs. Nonetheless, our results demonstrate that a short and non-disorder-focused course is feasible and is appreciated by participants. Initial experience and preliminary, exploratory results, interpreted with caution shown above, are promising and should soon be complemented by more comprehensive evaluations.

## Data Availability

The raw data supporting the conclusions of this article will be made available by the authors, without undue reservation.
