# Supplementary material for: HSN—helping in mental distress: a new emotion focused psychological first aid program
Source: Front Public Health. 2025 Jun 25;13:1589608. doi: 10.3389/fpubh.2025.1589608 (PMC12237678; doi:10.3389/fpubh.2025.1589608)
Supplement: Supplementary file 1 [file Table_1.DOCX]

Supplementary material

Figure 1 English translation of the German items used

| **To what extent do you agree with the following statements?** (Original: Wie sehr stimmst du folgenden Aussagen zu?)  Response scale: Strongly disagree - Somewhat disagree - Neutral - Somewhat agree - Strongly agree (Original: Stimme überhaupt nicht zu - Stimme eher nicht zu - Neutral - Stimme eher zu - Stimme vollkommen zu) | |
| --- | --- |
| **English translation** | **German original** |
| 1) I know what an ‘emotional crisis’ is. | 1) Ich weiß, was eine Seelische Notsituation ist. |
| 2) Meeting a person in mental distress, I know how to handle it. | 2) Wenn ich einer Person begegne, die sich in einer seelischen Notsituation befindet, dann weiß ich, wie ich damit umgehen kann. |
| 3) Meeting a person in mental distress, I can put myself in their place. | 3) Wenn ich einer Person begegne, die sich in einer seelischen Notsituation befindet, dann kann ich mich in ihre Situation/Befindlichkeit hineinversetzen. |
| 4) Meeting a person in mental distress, I observe them more closely. | 4) Wenn ich einer Person begegne, die sich in einer seelischen Notsituation befindet, dann beobachte ich sie etwas genauer. |
| 5) Meeting a person in mental distress, I can trust my gut feeling that something is off or unusual. | 5) Wenn ich einer Person begegne, die sich in einer seelischen Notsituation befindet, dann kann ich meinem Bauchgefühl „Da stimmt was nicht, da ist was komisch“ vertrauen. |
| 6) Meeting a person in mental distress, I can take good care of myself at the same time. | 6) Wenn ich einer Person begegne, die sich in einer seelischen Notsituation befindet, dann kann ich gleichzeitig gut auf mich achten. |
| 7) Meeting a person in mental distress, I can also be aware of my own feelings. | 7) Wenn ich einer Person begegne, die sich in einer seelischen Notsituation befindet, dann kann ich gleichzeitig meine eigenen Gefühle gut wahrnehmen. |
| 8) Meeting a person in mental distress, I know how to approach them and invite them to a conversation. | 8) Wenn ich einer Person begegne, die sich in einer seelischen Notsituation befindet, dann weiß ich, wie ich sie ansprechen und zu einem Gespräch einladen kann. |
| 9) Meeting a person in mental distress, I know how to refer them to another helper or support service. | 9) Wenn ich einer Person begegne, die sich in einer seelischen Notsituation befindet, dann weiß ich, wie ich sie an eine weitere Hilfeperson/-stelle übergeben kann. |
| 10) Meeting a person who expresses to me that they want to take their own life, I know what to do. | 10) Wenn ich einer Person begegne, die mir gegenüber äußert, sich das Leben nehmen zu wollen, weiß ich was zu tun ist. |
